# Prevalence and Associated Factors of Prediabetes and Diabetes in Rural and Peri-Urban Bangladesh: A Community-Based Cross-Sectional Study

**DOI:** 10.7759/cureus.87090

**Published:** 2025-07-01

**Authors:** Hasnat Sujon, Md Moshiur Rahman, Md Ahshanul Haque, Shakila Banu, Taiyaba Tabassum Ananta, Mohammad Habibur Rahman Sarker

**Affiliations:** 1 Technical Training Unit, icddr,b (formerly known as the International Centre for Diarrhoeal Disease Research, Bangladesh), Dhaka, BGD; 2 Graduate School of Biomedical and Health Sciences, Hiroshima University, Higashihiroshima, JPN; 3 Nutrition Research Division, icddr,b (formerly known as the International Centre for Diarrhoeal Disease Research, Bangladesh), Dhaka, BGD; 4 Graduate School of Innovation and Practice for Smart Society, Hiroshima University, Higashihiroshima, JPN

**Keywords:** bangladesh, community health worker, diabetes mellitus, dyslipidemias, mirzapur demographic surveillance system, prediabetes, prevalence, tangail district

## Abstract

Background and objectives

The global burden of prediabetes and diabetes is rising rapidly, particularly in low- and middle-income countries like Bangladesh, where lifestyle and nutritional transitions are accelerating. Economically disadvantaged rural populations often lack awareness and access to care, making them highly vulnerable. Measuring the prevalence and identifying associated factors is essential for guiding public health strategies and resource allocation. This study aimed to assess the prevalence and associated factors of prediabetes and diabetes in a rural and peri-urban Bangladeshi community to inform targeted prevention and control strategies.

Methodology

We recruited 891 adults for this cross-sectional study in January-June 2020 through random sampling from the Mirzapur Demographic Surveillance System in Bangladesh, ensuring at least five years of residency. Trained Community Health Workers collected sociodemographic data using a semi-structured questionnaire and performed physical examination and anthropometric measurements. We used fasting blood sugar to screen patients for prediabetes and diabetes, and measured serum creatinine and urine albumin to creatinine ratio to diagnose chronic kidney disease (CKD). We also collected blood samples to measure serum albumin, hemoglobin, total cholesterol, and triglycerides. Blood and urine samples were tested in an accredited laboratory.

Results

Prevalence of prediabetes was 38% (n=343), and diabetes was 17% (n=151). Age-specific prevalences of prediabetes were 5%, 12% and 22%, and of diabetes were 1%, 3% and 13%, for the age groups of 18-30, 31-45, and ≥46 years, respectively. Sex-specific prevalences of prediabetes were 22% and 17%, and diabetes were 9% and 8%, for female and male participants, respectively. In multiple multinomial logistic regression, for prediabetes, significant risk factor was age ≥31 years (adjusted odds ratio (aOR) 2.84, 95%CI 1.64-4.92), and protective factors were smokeless tobacco use (aOR 0.58, 95%CI 0.36-0.94) and hypoalbuminemia (aOR 0.48, 95%CI 0.24-0.99), and for diabetes, significant risk factors were age ≥31 years (aOR 4.04, 95%CI 1.37-11.95), abdominal obesity (aOR 2.80, 95%CI 1.49-5.30), CKD (aOR 2.80, 95% CI 1.70-4.62), hypercholesterolemia (aOR 1.97, 95%CI 1.17-3.30) and hypertriglyceridemia (aOR 2.33, 95%CI 1.44-3.79), and protective factors were being female (aOR 0.21, 95%CI 0.066-0.68), and smokeless tobacco user (aOR 0.32, 95%CI 0.17-0.63).

Conclusion

This study reveals a high burden of prediabetes and diabetes in a rural and peri-urban Bangladeshi population. The findings underscore the urgent need to strengthen community-based screening and targeted prevention strategies to address the growing diabetes burden in underserved areas.

## Introduction

Dubbed the ‘tsunami’ in global public health, the diabetes epidemic has disproportionately impacted South Asia, with a dramatic surge in prediabetes and diabetes cases over the past three decades. This alarming trend is driven by genetic susceptibility and epidemiological transitions stemming from lifestyle changes. The distinct South Asian phenotype, characterized by dysregulated metabolism and sarcopenic obesity, predisposes individuals to an earlier onset of diabetes and its complications, even at lower BMI levels compared to Caucasians. Additionally, South Asia, including Bangladesh, has undergone rapid economic transformation and urbanization since the 1990s [1].

Diabetes, a debilitating metabolic condition marked by chronic hyperglycemia due to faulty insulin production and/or action, has become one of the rapidly escalating global health crises of the 21st century [2] and the eighth leading cause of death and disability worldwide [3]. Prediabetes, on the other hand, represents an intermediate metabolic state between normoglycemia and diabetes. South Asia, home to one-quarter of the global population, grapples with an alarming 90.2 million individuals with diabetes, including 13.1 million in Bangladesh, which ranks eighth globally in diabetes prevalence [2]. Despite improvements in health indicators, Bangladesh's overburdened healthcare system is ill-equipped to effectively address this burden, leading to immense out-of-pocket healthcare expenditures [4]. Therefore, proactive preventive measures, such as early identification of prediabetes to initiate timely, cost-effective diabetes management, are imperative for countries like Bangladesh. Moreover, marginalized and economically disadvantaged populations, such as rural residents, exhibit less awareness of their glycemic status, heightened susceptibility to complications, and inadequate access to care [5], underscoring the necessity for periodic health assessments in these communities.

While extensive research on diabetes in Bangladesh exists, prediabetes remains largely underexplored. Prior studies have investigated diabetes prevalence and risk factors, but many are constrained by limited scope, such as focusing on specific age groups [6], urban populations [7], females [8], or lacking comprehensive sociodemographic data [6,8-13], laboratory findings [6,9-11], or rural-specific factors [6,8-12,14,15]. Studies that included prediabetes also exhibit similar limitations [11,12,15,16].

Using a random sample among rural and peri-urban adults from the Mirzapur Demographic Surveillance System (DSS), this study aimed to provide a more comprehensive and updated assessment of the prevalence and determinants of both prediabetes and diabetes in rural and peri-urban Bangladesh. In addition to sociodemographic data, we included detailed physical measurements and laboratory biomarkers, enabling us to evaluate both prevalence and associated clinical risk factors in a more comprehensive and integrated manner than prior studies. The findings are especially timely given the rising prevalence of non-communicable diseases in Bangladesh. By identifying modifiable risk factors, our study offers evidence to inform targeted public health interventions.

## Materials and methods

Study site, participants, and sample size

**Figure 1:**
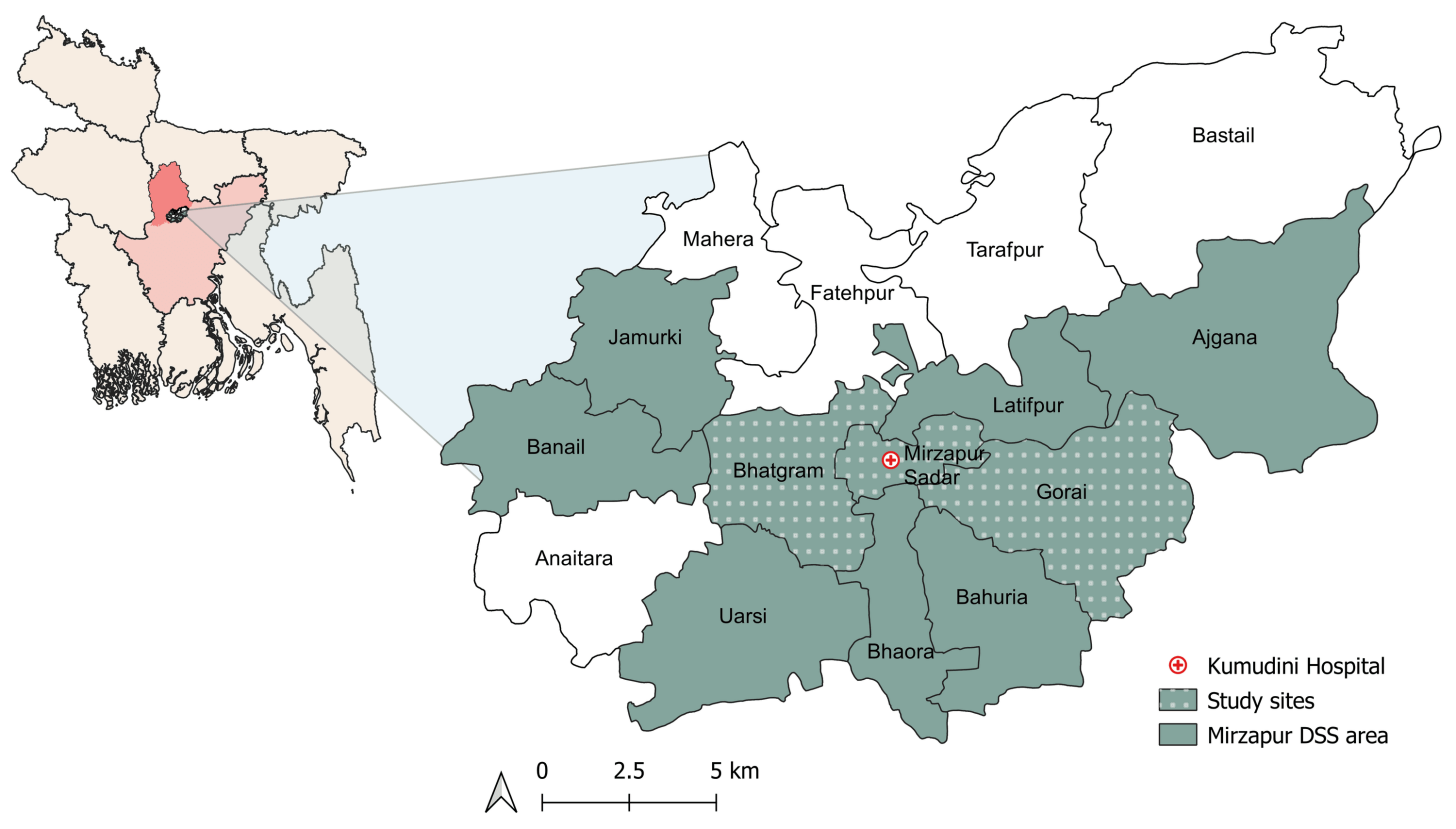
Mirzapur subdistrict of the Tangail district, Bangladesh. DSS: Demographic Surveillance System Image Credit: Hannah Leah Elbo Morito; permission has been provided for the use

The study was conducted as part of a larger study (ClinicalTrials.gov NCT04094831) [17]. This cross-sectional study was conducted from January to June 2020 in the Mirzapur subdistrict of the Tangail district in Bangladesh. Mirzapur, comprising 13 unions (smallest administrative division), with a mixed peri-urban and rural population, hosts the Mirzapur DSS, which has been collecting demographic data on approximately 300,000 residents of 10 unions since 2007. Due to resource constraints, we purposively selected three DSS unions (Mirzapur, Bhatgram, and Gorai) for this study (Figure 1). A total of 891 adults, residing in the DSS area for at least five years, were randomly selected. Individuals hospitalized during the study or with serious illnesses of uncertain prognosis, such as malignancy, mental illness, congenital disorders, or physical disabilities, were excluded.

Data collection

Data collection occurred in two phases. In the first phase, trained community health workers administered a pre-tested questionnaire [17] to collect sociodemographic data, including age, sex, education, occupation, marital status, family income, tobacco use, and sleeping duration. Anthropometric measurements (weight, height, waist, hip, and mid-upper arm circumference) and physical examinations (blood pressure) were also conducted. In the second phase, the researchers collected blood and urine samples at the Kumudini Hospital to assess the diabetes status, anemia, chronic kidney disease (CKD), and lipid profile. Diabetes was defined as a self-reported history of diabetes or fasting blood glucose ≥7 mmol/L; prediabetes as no diabetes history with fasting blood glucose between 5.6-6.9 mmol/L, and "no diabetes" as no diabetes history with fasting blood glucose <5.6 mmol/L [18].

Data analysis

Statistical analysis was performed using Stata 18 (StataCorp LLC, College Station, TX, USA). Although this was a cross-sectional study, diabetes (1) was treated as Case 1, prediabetes (2) as Case 2, and no prediabetes or diabetes (0) as the control, as outcome variables. All other variables were categorical and summarized as frequency and percentage, stratified by diabetes status. Simple multinomial logistic regression estimated unadjusted odds ratios (ORs) with “no prediabetes or diabetes” as the reference category. Multiple multinomial logistic regressions provided adjusted ORs (aORs) after controlling for all covariates in a single model. P-values and 95% confidence intervals (CI) were used for inferential estimates.

## Results

A total of 891 adults were included in the study. The study population predominantly comprised older adults, with the majority being female, married, and having some level of education. The prevalence of prediabetes and diabetes was 38% and 17%, respectively. Age-specific prevalence of prediabetes was 5%, 12%, and 22%, while diabetes prevalence was 1%, 3%, and 13% in the 18-30, 31-45, and ≥46 years age groups, respectively. Sex-specific prevalences of prediabetes and diabetes were higher in female participants (22% and 9%) compared to male participants (17% and 8%). Additional risk factors identified through physical and laboratory assessments are detailed in Table 1.

**Table 1:** Prevalence and associated factors of diabetes and prediabetes in Mirzapur, Tangail, Bangladesh among 18 years and older. OR: odds ratio; aOR: adjusted odds ratio; CI: confidence Interval; BMI: body-mass index; HDL: high density lipoprotein _^a^Prevalence of prediabetes was 38%._ _^b^Prevalence of diabetes was 17%._ _^c^Simple multinomial logistic regression was performed._ _^d^Multiple multinomial logistic regression was performed._ _^e^Age-specific prevalences of pre-diabetes were 5%, 12%, and 22% and diabetes were 1%, 3% and 13%, for the age group of 18–30, 31–45, and ≥46 years, respectively (N=891)._ _^f^Sex-specific prevalences of pre-diabetes were 22%, and 17%, and diabetes were 9%, and 8%, for females and males, respectively (N=891)._ _^g^Formal schooling of any level (at least primary education)._ _^h^Waist circumference ≥94 cm in males, and ≥80 cm in females._ _^i^Mid-upper arm circumference <25 cm in males, and <24 cm in females._ _^j^Currently requires antihypertensive therapy, and/or systolic blood pressure ≥140 mmHg, and/or diastolic blood pressure ≥90 mmHg._ _^k^Blood hemoglobin <13 g/dL in males, and <12 g/dL in females._ _^l^eGFR <60 ml/min/1.73 m2 or had albuminuria (ACR ≥30 mg/g)_ _^m^Serum total cholesterol >200 mg/dL._ _^n^Serum triglyceride >150 mg/dL._ _^o^Serum HDL-cholesterol <40 mg/dL._ _^p^­­­Serum albumin <3.5 g/dL._

Variables	Prediabetes^a^, n (%) (n=343)	Diabetes^b^, n (%) (n=155)	No prediabetes or diabetes, n (%) (n=393)	Total, n (%) (n=891)	Prediabetes				Diabetes			
					OR (95% CI)^c^	p-value	aOR (95% CI)^d^	p-value	OR (95% CI)^c^	p-value	aOR (95% CI)^d^	p-value
Age^e^ (years)												
18–30	42 (12)	6 (4)	95 (24)	143 (16)	Reference				Reference			
31-45	104 (30)	29 (19)	85 (22)	218 (25)	2.77 (1.74–4.39)	<0.01	2.84 (1.64–4.92)	<0.01	5.40 (2.14–13.64)	<0.01	4.04 (1.37–11.95)	0.01
≥46	197 (57)	120 (77)	213 (54)	530 (59)	2.09 (1.39–3.16)	<0.01	2.17 (1.21–3.88)	<0.01	8.92 (3.79–20.97)	<0.01	4.89 (1.66–14.38)	0.04
Sex^f^												
Male	150 (44)	75 (48)	167 (42)	392 (44)	Reference				Reference			
Female	193 (56)	80 (52)	226 (58)	499 (56)	0.95 (0.71–1.27)	0.74	0.73 (0.38–1.43)	0.36	0.79 (0.54–1.14)	0.21	0.21 (0.07–0.68)	<0.01
Formal education												
Literate^g^	213 (62)	104 (67)	246 (63)	563 (63)	0.98 (0.72–1.31)	0.89	0.92 (0.62– 1.35	0.68	1.22 (0.82- 1.81)	0.32	1.53 (0.91–2.56)	0.11
Occupation												
Farmer	28 (8)	13 (8)	38 (10)	79 (9)	0.76 (0.44–1.30)	0.32	0.85 (0.47–1.55)	0.60	0.82 (0.41–1.64)	0.57	0.67 (0.30–1.54)	0.35
Housewife	162 (47)	76 (49)	197 (50)	435 (49)	0.84 (0.63–1.15)	0.30	0.70 (0.38–1.26)	0.23	0.92 (0.62–1.36)	0.69	1.53 (0.51–4.60)	0.45
Marital status												
Married	270 (79)	130 (84)	303 (77)	703 (79)	1.52 (0.92–2.51)	0.10	0.86 (0.45–1.60)	0.28	3.28 (1.37–7.89)	<0.01	0.84 (0.27–2.62)	0.77
Widow	46 (13)	19 (12)	44 (11)	109 (12)	1.78 (0.95–3.34)	0.07	1.14 (0.51–2.55)	0.74	3.31 (1.21–9.06)	0.02	0.50 (0.13–1.86)	0.30
Family income												
≥$100/month	293 (85)	139 (90)	321 (82)	138 (15)	1.31 (0.88–1.96)	0.17	1.33 (0.86–2.04)	0.20	1.96 (1.09–3.44)	0.02	1.75 (0.90–3.44)	0.10
Sleeping duration												
<7 hours/24 hours	87 (25)	66 (43)	99 (25)	252 (28)	1.01 (0.72–1.41)	0.96	0.88 (0.60–1.29)	0.50	2.20 (1.49–3.26)	<0.001	1.55 (0.96–2.49)	0.06
Tobacco use												
Current tobacco smoker	62 (18)	19 (12)	93 (24)	174 (20)	0.71 (0.50–1.02)	0.06	0.78 (0.54–1.14)	0.20	0.45 (0.26–0.77)	<0.01	1.00 (0.60–1.66)	0.10
Current smokeless tobacco user	93 (27)	53 (34)	115 (29)	261 (29)	0.90 (0.65–1.24)	0.52	0.58 (0.36–0.94)	0.03	1.26 (0.84–1.87)	0.26	0.32 (0.17–0.63)	<0.01
Physical examination												
Underweight (BMI ≤18.5 kg/m^2^)	31 (9)	10 (6)	54 (14)	95 (11)	0.71 (0.44–1.15)	0.17	0.82 (0.43–1.56)	0.55	0.59 (0.28–1.21)	0.15	0.94 (0.35–2.58)	0.91
Overweight (BMI 25–29.9 kg/m^2^)	105 (31)	57 (37)	93 (23)	255 (29)	1.40 (0.99–1.96)	0.05	1.24 (0.78–1.99)	0.35	1.95 (1.28–2.97)	<0.01	0.81 (0.45–1.50)	0.51
Obesity (BMI ≥30 kg/m^2^)	22 (6)	16 (10)	17 (4)	55 (6)	1.60 (0.83–3.10)	0.16	1.33 (0.60–2.92)	0.48	2.99 (1.44–6.22)	<0.01	0.98 (0.38–2.57)	0.97
Abdominal obesity^h^	136 (40)	92 (59)	121 (31)	349 (39)	1.48 (1.09–2.00)	0.01	1.16 (0.72–1.88)	0.52	3.28 (2.23–4.83)	<0.01	2.80 (1.49–5.30)	<0.01
Undernutrition^i^	63 (18)	20 (13)	92 (23)	175 (20)	0.74 (0.51–1.05)	0.09	1.00 (0.60–1.66)	0.99	0.48 (0.29–0.82)	<0.01	1.49 (0.67–3.30)	0.33
Hypertension^j^	140 (41)	94 (61)	131 (33)	365 (41)	1.38 (1.02–1.86)	0.04	1.12 (0.79–1.60)	0.52	3.08 (2.10–4.53)	<0.001	1.14 (0.71–1.84)	0.56
Laboratory investigations												
Anemia^k^	79 (24)	30 (19)	86 (22)	203 (23)	1.21 (0.86–1.71)	0.27	1.42 (0.97–2.08)	0.07	0.86 (0.54–1.36)	0.51	0.65 (0.37–1.13)	0.13
Chronic kidney disease^l^	77 (22)	77 (52)	89 (23)	245 (27)	1.07 (0.75–1.51)	0.76	0.86 (0.58–1.29)	0.47	3.76 (2.52–5.61)	<0.001	2.80 (1.70–4.62)	<0.001
Hypercholesterolemia^m^	117 (34)	55 (35)	59 (15)	191 (21)	1.64 (1.12–2.38)	0.01	1.37 (0.91–2.06)	0.14	3.11 (2.02–4.79)	<0.001	1.97 (1.17–3.30)	0.01
Hypertriglyceridemia^n^	113 (33)	85 (55)	96 (24)	298 (33)	1.60 (1.16–2.20)	<0.01	1.39 (0.95–2.01)	0.09	3.76 (2.54–5.55)	<0.001	2.33 (1.44–3.79)	<0.01
Low HDL-cholesterol^o^	14 (4)	61 (39)	124 (32)	298 (33)	1.06 (0.78–1.45)	0.69	0.82 (0.57–1.19)	0.30	1.41 (0.96–2.07)	0.08	1.02 (0.62–1.69)	0.93
Hypoalbumenemia^p^		17 (11)	27 (7)	58 (7)	0.58 (0.30–1.12)	0.10	0.48 (0.24–0.99)	0.05	1.67 (0.88–3.16)	0.12	1.66 (0.77–3.59)	0.20

Simple multinomial logistic regression identified several factors to be associated with prediabetes and diabetes. Significant risk factors of prediabetes were age ≥31 years, overweight, abdominal obesity, hypertension, hypercholesterolemia, and hypertriglyceridemia. Significant risk factors of diabetes were age ≥31 years, being married, widowed, family income ≥$100/month, sleeping <7 hours/24 hours, overweight, obesity, abdominal obesity, hypertension, CKD, hypercholesterolemia, and hypertriglyceridemia. Current tobacco smoking and undernutrition were identified as protective factors against diabetes (Table 1).

In multiple multinomial logistic regression, significant risk factors for prediabetes included age 31-45 years and ≥46 years, while smokeless tobacco use and hypoalbuminemia were protective factors. For diabetes, significant risk factors were age 31-45 years and ≥46 years, abdominal obesity, CKD, hypercholesterolemia, and hypertriglyceridemia, while being female and smokeless tobacco user were protective factors (Table 1).

## Discussion

In our study, prediabetes and diabetes prevalences were 38% and 17%, respectively, with older adults exhibiting the highest age-specific rates, and females demonstrating the highest sex-specific rates. Age, elevated BMI, abdominal obesity, hypertension, hypercholesterolemia, and hypertriglyceridemia were risk factors for both conditions.

The observed prevalences in our sample exceeded national averages, which range from 12% to 32% for prediabetes and 8% to 9% for diabetes [11,12,15] in rural Bangladesh. A greater representation of female and older individuals in the current study might be the reason for this discrepancy. However, our sole reliance on fasting blood glucose for diagnosing prediabetes and diabetes typically yields a lower prevalence than combined diagnostic criteria (fasting blood glucose, two-hour post-prandial blood glucose, and HbA1c) [18], indicating an upward trend in these conditions among rural residents. Consistent with prior studies, we observed that the prevalence of both prediabetes and diabetes increases with age [9-12,14,15]. While diabetes prevalence by sex is consistent with prior studies, prediabetes prevalence differs [9-12,14,15], with female participants showing higher rates [11,12,15]. As in previous studies, female populations are more prone to diabetes and prediabetes than male populations [11,12,15]. The International Diabetes Federation projects that Bangladesh will rank seventh globally in diabetes cases by 2045 [2]. Our finding that over one-third of rural residents have prediabetes aligns with this projection, indicating an impending strain on Bangladesh’s healthcare system if urgent preventive measures are not implemented. This rapid increase in the prevalence may stem from evolving risk factors, particularly socioeconomic changes, as discussed below. Bangladesh is also acknowledged to be going through the third stage of the epidemiological transition from acute, infectious, and parasitic diseases to non-communicable diseases [19].

Advancing age is a well-recognized, non-modifiable risk factor for both prediabetes and diabetes [20]. Our findings indicate that individuals aged ≥46 years have a 2.17-fold higher risk of developing prediabetes and a 4.89-fold higher risk of developing diabetes compared to the 18-30 year age group. Closely monitoring prediabetes in the older population is crucial, as 40% of people with prediabetes progress to overt diabetes [21], and microvascular complications can arise even before the onset of frank hyperglycemia. The age-related increase in susceptibility to prediabetes and diabetes is attributed to declining insulin secretion, compromised pancreatic function, and heightened insulin resistance [20]. With rising life expectancy and a concerning conversion rate from prediabetes to diabetes, Bangladesh is poised to face a substantial diabetes burden in the near future.

Another common risk factor for both diabetes and prediabetes was higher BMI and abdominal obesity, although this association was attenuated in the multiple multinomial analysis, likely due to multicollinearity. Previous studies have demonstrated a concurrent rise in the prevalence of diabetes alongside increasing overweight and obesity [10,22]. While examining the links between excess weight and these metabolic conditions, it is crucial to consider the unique body fat composition of South Asians and its implications for disease patterns. Compared to Caucasian individuals, South Asian individuals exhibit a higher waist-to-hip ratio and greater total and abdominal fat despite lower BMI. Due to these ethnic differences, abdominal obesity shows a stronger relationship with glycemic control than BMI in this population. Furthermore, South Asian people have thicker subcutaneous fat deposits associated with metabolic syndrome. This body fat distribution, combined with relatively diminished muscle mass, facilitates a leaner body habitus that paradoxically heightens susceptibility to metabolic dysregulation. Recognizing these ethnic-specific trends, guidelines in India, Sri Lanka, and the United Kingdom now recommend distinct BMI thresholds for South Asian populations: >18 kg/m^2^ for underweight, 18-<23 kg/m^2^ for normal weight, 23-<25 kg/m^2^ for overweight, and ≥25 kg/m^2^ for obesity [1].

The genetic predisposition to adverse metabolic consequences is further compounded by the high-glycemic diet and sedentary lifestyle prevalent in this region. Therefore, the association between excess weight and prediabetes/diabetes is multifaceted, also involving socioeconomic status, physical activity, occupation, and dietary patterns. Mirroring broader trends in South Asia, Bangladesh has undergone rapid urbanization in recent decades, driving corresponding shifts in lifestyle behaviors. As reported in previous studies [9], our findings indicate that higher monthly family income is a risk factor for diabetes, though it did not appear to influence the risk of prediabetes. While we did not investigate dietary patterns in this study, the ongoing process of urbanization has led more individuals to transition from manual labor to white-collar jobs, with greater access to fast foods high in refined carbohydrates and sugars [1,23]. Traditional cuisines, on the other hand, contain high omega-6 and low omega-3 fatty acid ratios that increase the risk of diabetes, are cooked in oil and fats, and often involve cooking methods like prolonged heating that can convert healthier unsaturated fats into harmful trans fats [1]. Prior research has established that the rising prevalence of overweight and obesity is the primary driver of the diabetes epidemic in Bangladesh [6]. Given that moderate weight loss can effectively reduce the incidence of diabetes [24], public health strategies aimed at diabetes prevention in this country should prioritize tackling the obesity crisis.

Our findings reveal a concerning prevalence of hypertension among individuals with prediabetes and diabetes (61% and 41%, respectively), which exceeds national averages [11]. A higher representation of the older population in our sample might be the reason behind this. Importantly, hypertension has been consistently identified as a significant risk factor for both prediabetes and diabetes, as in previous studies [11,25], although its effect did not reach statistical significance in our multiple multinomial analysis. The relationship between hypertension and these metabolic conditions is complex, as they share common physiological traits [26], and both heighten the risk of cardiovascular complications [27]. Furthermore, hypertension is exacerbated by factors such as advanced age, excess weight, poor dietary habits, physical inactivity, and dyslipidemia- patterns that mirror the drivers of prediabetes and diabetes. Given the high prevalence of comorbid hypertension, urgent public health measures are warranted to tackle this alarming situation.

Our findings indicate that both elevated cholesterol and high triglyceride levels are significant risk factors for developing diabetes. A similar pattern was observed for prediabetes, though the association was not statistically significant in the multiple multinomial analysis. Alarmingly, we also noted a high prevalence of hypertriglyceridemia among individuals with diabetes. Abnormal lipid profiles have been well-documented in Bangladeshi populations with diabetes [28]. This is particularly concerning because high triglycerides and low high-density lipoprotein (HDL) cholesterol are common among South Asians, and the cardiovascular protective effects of high HDL-cholesterol are relatively lower in this group [28]. Dyslipidemia increases the risk of both macrovascular and microvascular complications of diabetes [29], and patients with diabetes tend to benefit more from lipid-lowering therapies compared to those without the condition [30].

Our findings indicate that individuals with CKD have a 2.8-fold higher risk of developing diabetes, although no such association was observed for prediabetes. The relationship between diabetes and CKD is bidirectional. Diabetes is a well-established risk factor for CKD, which can progress to diabetic nephropathy and end-stage renal disease. Conversely, impaired kidney function can also compromise glucose metabolism, leading to insulin resistance and diabetes. Given the alarmingly high prevalence of CKD among those with prediabetes (24%) and diabetes (52%), there is an urgent need to aggressively address these two interrelated conditions through concerted public health efforts.

Although being married or widowed and sleeping less than seven hours a day emerged as risk factors for diabetes in univariate analysis, they did not reach significance in multiple multinomial analysis for prediabetes. Nonetheless, evidence suggests that inadequate and irregular sleep is as critical as obesity and physical inactivity in contributing to diabetes, given its essential role in maintaining endocrine and metabolic functions. Sleep disturbances from long work hours, nocturnal light exposure, and irregular mealtimes exacerbate this risk [1]. Despite this, the link between insufficient sleep and diabetes requires further investigation. Contrary to previous studies [1], our findings revealed that current tobacco smoking significantly protects against diabetes, and smokeless tobacco use significantly protects against prediabetes. While cigarette smoking is a global public health threat and smokeless tobacco use is rampant in South Asia, particularly in Bangladesh, these unexpected protective effects warrant further exploration. Additionally, undernutrition appeared protective against diabetes, and hypoalbuminemia against prediabetes, highlighting the need to delve deeper into these findings within this rural community. The protective effect of smokeless tobacco use, undernutrition, and hypoalbuminemia may reflect unmeasured confounding or population-specific behavior rather than a causal relationship and should be interpreted with caution.

Our study suggests that prediabetes and diabetes rates in rural Bangladesh may be rising faster than previously thought. This potential crisis demands urgent action from all stakeholders to implement aggressive preventive strategies. Among the modifiable risk factors, weight gain is a primary driver of this increase. Therefore, promoting healthy diets and physical activity should be central to prevention efforts [23]. Raising community awareness and encouraging widespread participation by supporting access to nutritious food and opportunities for physical activity are crucial steps. In addition, healthcare professionals have an important role to play. It has been observed that there is clinical inertia among healthcare professionals regarding lifestyle advice where medication is available. While drugs may be essential to manage diabetes, and people with diabetes can enormously benefit from lipid-lowering agents where necessary, these are not replacements for lifestyle modification [23]. As Bangladesh is not currently well-placed to address its diabetes burden, a reappraisal of the current capabilities would be a welcome step. The government’s current non-communicable disease screening program has been criticized for inconsistency, high age bar, and limited coverage [9]. To improve outcomes, revising screening and management strategies and incorporating culturally sensitive innovations, such as mHealth support [31], are recommended to maximize coverage and effectiveness.

Strengths and limitations

Random sampling with a large sample size, the use of an accredited laboratory, and validated and standardized equipment were the main strengths of the study. The cross-sectional design, convenient selection of three unions, and the study being limited to one subdistrict, which may not project the general picture of the entire country, were the main limitations of the study. We solely relied on fasting blood sugar for detecting diabetes status, which risks missing cases with impaired glucose tolerance and diabetes cases. Additionally, crucial variables like food intake and physical activity levels were out of the scope of the study, which could have provided a more comprehensive understanding of the factors influencing prediabetes and diabetes.

## Conclusions

We have provided a recent update on the prevalence and risk factors of prediabetes and diabetes in rural Bangladesh in this study. The prevalence, along with multiple comorbidities is alarmingly high among rural adults. Given the economic marginalization of this population, there is a heightened risk of these comorbidities becoming complicated and necessitating costly interventions. Therefore, a sustainable, community-wide approach is essential to integrate effective preventive and management strategies into daily life. Immediate action is also needed to expand screening and management coverage in rural Bangladesh to address this urgent public health issue.
